# Steroid-Responsive, Post-infectious New Daily Persistent Headache With Mild Cerebrospinal Fluid Pleocytosis: A Case Report

**DOI:** 10.7759/cureus.102843

**Published:** 2026-02-02

**Authors:** Shoji Kikui, Yoshiki Matsumoto, Daisuke Danno, Takao Takeshima

**Affiliations:** 1 Neurology, Tominaga Hospital, Osaka, JPN

**Keywords:** 3rd edition, international classification of headache disorders, new daily persistent headache, post-infectious, steroid

## Abstract

New daily persistent headache (NDPH) is a primary headache disorder characterized by a clearly remembered onset, with the headache becoming daily and unremitting within 24 hours and persisting for more than 3 months. Although antecedent infections have been reported as potential triggering events, the pathophysiology of post-infectious presentations of NDPH remains incompletely understood, and effective treatment options are limited. We report the case of a 42-year-old man who developed a continuous daily headache following a flu-like illness and was diagnosed with NDPH according to the International Classification of Headache Disorders, 3rd edition. Cerebrospinal fluid examination revealed mild pleocytosis, while neurological examination and neuroimaging findings were unremarkable. Intravenous methylprednisolone therapy, followed by a short course of oral corticosteroids, resulted in rapid and sustained complete resolution of the headache. This case suggests that inflammatory or immune-related mechanisms may contribute to headache persistence in a subset of patients with NDPH triggered by antecedent infection. However, responsiveness to corticosteroid therapy does not necessarily indicate the presence of ongoing active inflammation and may instead reflect reversible modulation of mechanisms involved in headache chronification. Further studies are required to clarify the pathophysiology of post-infectious NDPH and to identify patients who may benefit from targeted therapeutic approaches.

## Introduction

New daily persistent headache (NDPH) is a headache disorder characterized by a clearly remembered onset, with headache becoming daily and unremitting within 24 hours and persisting for more than 3 months. First described in 1986 and later reviewed historically by Robbins et al. in an interview with Vanast [1], NDPH is currently classified as an “other primary headache disorder” in the International Classification of Headache Disorders, 3rd edition (ICHD-3) [2]. Although an abrupt onset is a defining feature, the clinical phenotype of NDPH is heterogeneous. Patients may present with migraine- or tension-type headache-like features, making differentiation from chronic migraine or chronic tension-type headache challenging in clinical practice [2,3]. Despite its relatively low prevalence, NDPH is often highly disabling and frequently resistant to conventional headache treatments.

Various triggering events have been reported in association with NDPH, including infections, stressful life events, and surgical procedures [4]. In particular, a substantial proportion of patients describe headache onset following a flu-like or infectious illness, suggesting that antecedent infection may act as a triggering factor in some cases [4,5]. These observations support the concept that NDPH represents a heterogeneous syndrome rather than a single disease entity.

Accumulating evidence supports the existence of post-infectious NDPH [5-7]. These findings raise the possibility that inflammatory or immune-related mechanisms may contribute to headache persistence in some patients with NDPH.

Here, we report a case of NDPH that developed after a flu-like illness, was accompanied by mild cerebrospinal fluid (CSF) pleocytosis, and showed a rapid and sustained response to corticosteroid therapy.

## Case presentation

A 42-year-old Japanese man presented with influenza-like symptoms, including a high fever of approximately 38 °C and a sore throat. These symptoms persisted for approximately one week and resolved spontaneously without treatment. After the systemic symptoms had completely resolved, the patient experienced the sudden onset of a diffuse, pulsatile headache involving the entire head, which became continuous and unremitting within 24 hours. The headache occurred daily and was moderate to severe in intensity. He had no prior history of episodic headache, and his past medical and family histories were unremarkable. The headache persisted without remission thereafter.

Approximately two months prior to admission, the patient was evaluated by a general practitioner. Brain magnetic resonance imaging (MRI) and magnetic resonance angiography (MRA) revealed no structural abnormalities. He was diagnosed with tension-type headache and treated with etizolam, tizanidine, and diclofenac; however, these treatments were ineffective. Because the headache was refractory and persistent, he was referred to our department and was admitted for further evaluation and treatment three months after headache onset.

On admission, his height and weight were 164 cm and 60 kg, respectively. Vital signs were stable, with a body temperature of 36.3 °C, blood pressure of 122/82 mmHg, and heart rate of 76 beats per minute. He was fully alert, and general physical and neurological examinations revealed no focal neurological deficits. The headache was continuous, diffuse, and pulsatile, with a numerical rating scale score of 6-8. There was no postural component to the headache, and the patient denied nausea, vomiting, photophobia, phonophobia, or cranial autonomic symptoms. No neck stiffness was observed.

Laboratory tests, including complete blood count, serum biochemistry, electrolytes, and inflammatory markers, were all within normal limits. Both C-reactive protein and erythrocyte sedimentation rates were normal. Thyroid function tests and routine autoimmune serological screening revealed no abnormalities in the patient. Screening tests for infectious diseases, including syphilis, hepatitis B, hepatitis C, and human immunodeficiency virus, were all negative. Serological testing for herpes simplex virus and varicella-zoster virus showed no significant elevation of immunoglobulin G or M titers.

Cerebrospinal fluid (CSF) examination revealed an opening pressure of 120 mmH₂O. The CSF was clear and colorless, with a mildly elevated cell count of 20 cells/mm³ (18 mononuclear cells and 2 polymorphonuclear cells). The CSF glucose (55 mg/dL) and protein (41.0 mg/dL) levels were within normal limits. The CSF immunoglobulin (IgG) index was also normal. CSF cultures and cytology were negative, and no evidence of tuberculosis was found. Based on the patient’s age, clinical presentation, contrast-enhanced MRI findings, cerebrospinal fluid analysis, and epidemiological timeline, major secondary causes of steroid-responsive headache were systematically evaluated and excluded (Table 1).

**Table 1:** Evaluation for secondary causes of steroid-responsive headache Systematic evaluation of major secondary causes of steroid-responsive headache based on clinical features, contrast-enhanced neuroimaging, cerebrospinal fluid analysis, laboratory findings, and epidemiological considerations. The table summarizes the diagnostic tests performed, key results, and conclusions supporting the exclusion of inflammatory, autoimmune, infectious, and post–COVID-19 conditions. MRI: magnetic resonance imaging, CSF: cerebrospinal fluid, IgG: immunoglobulin G, COVID-19: coronavirus disease 2019

Condition considered	Diagnostic tests performed	Results	Conclusion
Giant cell arteritis	Assessment of age at onset, clinical features, inflammatory markers	Onset in the 40s; no cranial ischemic symptoms; inflammatory markers within normal limits	Unlikely
Hypertrophic pachymeningitis	Contrast-enhanced brain MRI	No dural thickening or enhancement	Excluded
IgG4-related disease	Clinical assessment; contrast-enhanced brain MRI; routine laboratory tests	No organ enlargement or multi-organ involvement; no dural thickening on MRI; serum IgG4 not measured	Unlikely
Autoimmune encephalitis/meningitis	Neurological examination; brain MRI; CSF analysis, including IgG index	Headache only without encephalopathy, seizures, or focal deficits; MRI unremarkable; CSF IgG index within normal limits	Unlikely
Central nervous system infection	Clinical assessment; CSF analysis, including culture and cytology	No fever or altered mental status; CSF culture and cytology negative	Unlikely
Long COVID–related headache	Review of epidemiological timeline and clinical history	Headache onset preceded the COVID-19 pandemic	Unlikely

**Figure 1:**
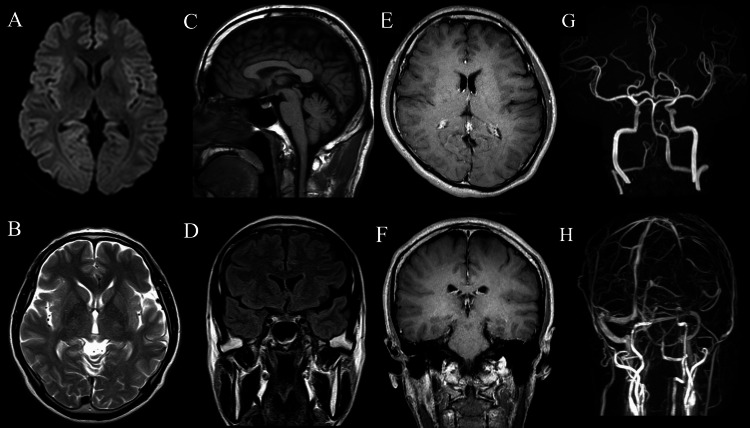
Brain MRI, MRA, and MRV findings Diffusion-weighted imaging shows no acute ischemic lesions (A); Axial T2-weighted imaging reveals no parenchymal abnormalities (B); Sagittal T1-weighted imaging demonstrates normal brain morphology without evidence of structural lesions (C); Coronal fluid-attenuated inversion recovery (FLAIR) imaging shows no abnormal signal intensity (D); Contrast-enhanced axial T1-weighted imaging shows no abnormal enhancement (E); Contrast-enhanced coronal T1-weighted imaging reveals no pathological enhancement (F); Magnetic resonance angiography (MRA) demonstrates no intracranial arterial abnormalities (G); Magnetic resonance venography (MRV) reveals no evidence of cerebral venous sinus thrombosis or venous outflow obstruction (H)

Additional investigations, including chest radiography, electrocardiography, brain MRI, MRA, and magnetic resonance venography (MRV), revealed no abnormalities (Figure 1). Based on a clearly remembered onset, the development of daily persistent headache within 24 hours, and persistence of symptoms for more than three months, the patient met the diagnostic criteria for NDPH according to the International Classification of Headache Disorders, 3rd edition (ICHD-3) [2]. Given the temporal association with a preceding influenza-like illness and the presence of mild CSF pleocytosis, the condition was considered post-infectious NDPH.

Based on the patient’s informed consent, intravenous methylprednisolone pulse therapy (1,000 mg/day) was administered for three days. During treatment, the headache rapidly and completely resolved. Subsequently, oral prednisolone was initiated at 60 mg/day and gradually tapered over approximately two weeks. During follow-up in the outpatient setting, the patient remained free of headache symptoms, with no recurrence observed.

## Discussion

The present case fulfilled the diagnostic criteria for NDPH according to the ICHD-3, based on a clearly remembered onset, rapid transition to a continuous daily headache within 24 hours, and persistence of symptoms for > 3 months [2]. However, the clinical course, characterized by a preceding flu-like illness, mild CSF pleocytosis, and a favorable response to corticosteroid therapy, was atypical for classic primary NDPH and warrants careful clinical interpretation.

Although NDPH is classified as a primary headache disorder in the ICHD-3, accumulating evidence suggests that it represents a heterogeneous condition with multiple potential triggering factors, including infections, stressful life events, and surgical procedures [2-4]. In particular, antecedent infectious illnesses have frequently been reported in patients with NDPH, supporting the concept of a post-infectious form described in the literature [5-7]. However, “post-infectious NDPH” is not formally recognized as a distinct diagnostic entity in the ICHD-3. In the present case, the diagnosis of NDPH was made strictly according to ICHD-3 criteria, and the term “post-infectious” is used descriptively to indicate a probable triggering event rather than to imply a secondary headache disorder.

The mild CSF pleocytosis observed in this patient raises the possibility that inflammatory or immune-related mechanisms may contribute to headache persistence in a subset of NDPH cases. However, interpretation of this finding warrants caution. CSF examination was performed approximately three months after headache onset and therefore does not reflect acute-phase inflammatory changes. As a result, the presence or absence of meningitis at symptom onset cannot be definitively determined. Moreover, neurological examination and neuroimaging findings remained unremarkable throughout the clinical course.

Viral meningitis was considered in the differential diagnosis given the antecedent flu-like illness and mild CSF pleocytosis. However, viral meningitis is typically self-limited, with headache and systemic symptoms resolving within days to weeks as inflammation subsides [8]. By contrast, the present patient developed a continuous and unremitting headache persisting for more than three months. Chronic meningitis was also considered; however, it is usually characterized by persistently abnormal CSF findings, progressive neurological symptoms, and identifiable infectious or non-infectious etiologies, including tuberculosis, fungal infection, malignancy, sarcoidosis, and autoimmune disease [9]. The absence of progressive symptoms, normal neuroimaging findings, and rapid and sustained symptom resolution following corticosteroid therapy without relapse argues against active chronic meningitis in this case.

A notable feature of the present case was the rapid and sustained response to corticosteroid treatment. Previous reports, including the case series by Prakash and Shah, have described the improvement of NDPH-like headache following corticosteroid treatment in selected patients with post-infectious onset [7]. However, these observations were derived from small, uncontrolled studies, and corticosteroid responsiveness cannot be considered a defining or generalizable feature of NDPH.

Importantly, NDPH is a descriptive clinical phenotype rather than a biological diagnosis, and a favorable response to corticosteroid therapy alone does not establish a specific underlying mechanism. In the present case, major secondary causes of steroid-responsive headache, including inflammatory, autoimmune, and infectious disorders, were systematically evaluated and excluded based on contrast-enhanced MRI findings, cerebrospinal fluid analysis, laboratory data, and epidemiological considerations (Table 1). Therefore, the observed corticosteroid responsiveness should be interpreted as a clinical characteristic of this patient rather than as evidence of an underlying inflammatory disease.

From this perspective, the present case may be best interpreted as NDPH triggered by a probable infectious event, with subsequent headache persistence driven by mechanisms similar to those underlying primary NDPH as defined in the ICHD-3. The observed corticosteroid responsiveness may therefore reflect reversible modulation of these mechanisms rather than treatment of an ongoing secondary inflammatory disorder.

This case underscores the importance of careful clinical evaluation in patients presenting with abrupt-onset daily headaches. Although NDPH is classified as a primary headache disorder, clinicians should remain alert to possible post-infectious or immune-related mechanisms, particularly in patients with a clear infectious trigger or subtle CSF abnormalities. Given the limitations inherent in single case reports and the absence of acute-phase data, corticosteroid therapy should be considered cautiously and on an individual basis. Further studies are needed to clarify the pathophysiological mechanisms underlying post-infectious presentations of NDPH and to identify clinical or biological markers that may predict treatment responsiveness.

## Conclusions

This case describes a steroid-responsive presentation of NDPH following a flu-like illness, accompanied by mild CSF pleocytosis and a favorable clinical outcome. Although NDPH is classified as a primary headache disorder, it represents a descriptive clinical phenotype rather than a diagnosis based on the underlying biological mechanisms. In this case, responsiveness to corticosteroid therapy should be interpreted as a clinical observation and does not imply the presence of an ongoing, secondary inflammatory disorder. Further studies are warranted to clarify the clinical heterogeneity of NDPH and identify the factors associated with treatment response.
